# Editorial on Genomic Mosaicism in Human Development and Diseases

**DOI:** 10.3390/genes16060697

**Published:** 2025-06-09

**Authors:** Xincen Xi, Xiaoxu Yang

**Affiliations:** Department of Human Genetics, University of Utah, Salt Lake City, UT 84112, USA; xincen.xi@utah.edu

The individual genome continuously accumulates genetic variations from a zygote [1]. Despite their effective DNA repair mechanisms, many of these variants remain in the genome and are passed down to daughter cells [2]. This leads to the presence of distinct genomic sequences among different cells, tissues, and organs within the same individual, a phenomenon known as genomic mosaicism [3,4]. Genomic mosaicism arises from post-zygotic mutations and can occur during embryonic development, tissue self-renewal, environmental exposure to toxicity, and aging. It is reported in more than 250 different human diseases, including various cancers [5,6]. While most mosaic mutations remain confined to somatic cells, germline mosaicism can affect reproductive cells, leading to the transmission of these mutations to the next generation [7]. In this Special Issue “Genomic Mosaicism in Human Development and Diseases”, we present six publications on this topic from different angles.

In the review by Geiger et al. from Vanderbilt University, the authors summarized the clinical spectrum of mosaic genetic diseases [8]. The authors reviewed mosaic genetic diseases across the spectrum of phenotypes: Mild mosaicism has little or no phenotypic effects on the individual, but increases the transmission risk of a pathogenic genotype to the next generation [9]. Moderate mosaicism can reduce the phenotype severity of genetic disease or the full prevalence of disorders. Severe mosaicism is observed in conditions that are typically lethal in non-mosaic individuals, but where mosaicism allows for survival. This provides a comprehensive perspective on the impact of genomic mosaicism on human development and related diseases. One example of the transmissible mosaic mutation in the male germline is presented in a research article by Striedner et al., to explore the micro-mosaic landscape of FGFR3 variants in the aging male germline. The study specifically examines two FGFR3 variants—c.1138G>A (p.G380R), associated with achondroplasia (ACH), and c.1948A>G (p.K650E), linked to thanatophoric dysplasia type II (TDII)—to investigate their expansion patterns in the testis and transmission into sperm. The authors found that, unlike the achondroplasia variants that showed sub-clonal expansion events in aged testes and a significant increase in mutant sperm with the donor’s age, the thanatophoric dysplasia variants showed focal mutation pockets in the testis, but exhibited reduced transmission into sperm and no significant age-related increase. The divergence in transmission patterns suggests that the mutant receptor tyrosine kinase (RTK) activity may influence male germline differentiation, particularly in meiosis-dependent processes or selective expansion events.

Mosaicism by nature can involve any type of genomic mosaic variation ranging from mosaic single-nucleotide variants or small insertion deletions, mosaic short tandem repeats, or somatic transpositions, all the way up to mosaic copy number variation, structural variations, and mosaic chromosomal abnormalities [10]. In the review presented by Doss et al., the authors introduced how short tandem repeat variants in the germline and somatic tissue are causing human disorders, especially in mosaic format [11]. The review focused on three relatively well-studied examples, i.e., Fragile X, Hungtington’s disease, and Myotonic Dystrophy Type I. The unstable expansion of STR fragments in these diseases may lead to differences in repeat copy numbers between different cell types, resulting in phenotypic variation. This instability in expansion not only varies across different tissues and cell types within an individual, but also between individuals in terms of somatic STR mosaic patterns. Consequently, this variability may cause significant differences in disease severity, age of onset, and clinical manifestations among patients. It demonstrated how critical the role of mosaicism is both for cellular physiology and clinical phenotypes. For larger mosaic variants such as chromosomal trisomies, Kovaleva and Cotter presented a comprehensive research article of 1266 published cases focusing on maternal age and reproductive history. They found a higher occurrence of a previous pregnancy with a mosaic chromosome abnormality, namely 1/13 in the prenatal cohort and 1/16 in the postnatal cohort, which is five-fold higher compared to published studies on non-mosaic trisomies, implying that maternal reproductive history influences the occurrence of mosaic trisomy. Further analysis revealed a positive correlation between maternal age (≥35 years) and the risk of mosaic trisomy, indicating that older mothers are more likely to conceive a fetus with mosaic trisomy.

From a clinical point of view, the fast-advancing sequencing technologies and variant detection and interpretation methodologies empowered by machine learning and artificial intelligence tools are significantly advancing our understanding of developmental disorders caused by genomic mosaicism [12,13]. The research article by Vado et al. from Araba University Hospital talked about their practices in the sampling and genetic testing of Fibrous Dysplasia/McCune-Albright syndrome in a clinical cohort of 40 patients with Sanger sequencing, where they observed significantly improved detection rates with Next-Generation Sequencing. They found that it was essential to use the more sensitive techniques to allow for the detection of low-fraction variants and recommended that patients with these disorders be genetically tested to facilitate clinical diagnosis. The study on Focal Cortical Dysplasia type III presented by Garcia et al. reported 10 novel mutations for this disease after employing machine-learning-based variant detection and functional prediction methodologies in 19 patients diagnosed with Focal Cortical Dysplasia type III. These variants have a severe destabilizing effect on protein structure and are also related to clinical manifestations such as encephalopathies and intellectual disabilities. Notably, these mosaic variants are not enriched on the mTOR pathway, which has been previously implicated in other subtypes of Focal Cortical Dysplasia [14], and are predicted to cause developmental phenotypes with a similar degree of deleterious mutation.

In summary, this Special Issue provides a thorough view of how genomic mosaicism is related to human development and related diseases and underscores how additional large-scale efforts need to be made for the systematic study of further clinical implications.
